# Supplementary material for: A Frailty-Adjusted Stratification Score to Predict Surgical Risk, Post-Operative, Long-Term Functional Outcome, and Quality of Life after Surgery in Intracranial Meningiomas
Source: Cancers (Basel). 2022 Jun 22;14(13):3065. doi: 10.3390/cancers14133065 (PMC9265059; doi:10.3390/cancers14133065)
Supplement: Supplementary file 1 [file cancers-14-03065-s001.zip › cancers-1716671-supplementary.pdf]

Table S1. Cumulative risk chart.

Milan Biometric Surgical Score - Part A	Frequency (N)	RR	RR 95% C.I.	
0	16	2,611	1,469	4,640
1	4	5,222	2,938	9,280
2	25	7,833	4,407	13,920
3	22	10,444	5,876	18,560
4	18	13,055	7,345	23,200
5	23	15,666	8,814	27,840
6	9	18,277	10,283	32,480
7	16	20,888	11,752	37,120
8	13	23,499	13,221	41,760
9	7	26,110	14,690	46,400
10	4	28,721	16,159	51,040
11	3	31,332	17,628	55,680
12	4	33,943	19,097	60,320
13	1	36,554	20,566	64,960
14	1	39,165	22,035	69,600
15	0	/	/	/
**Milan Biometric Surgical Score - Part B**				
0	10	2,961	1,988	4,411
1	27	5,572	3,457	9,051
2	39	8,183	4,926	13,691
3	34	10,794	6,395	18,331
4	26	13,405	7,864	22,971
5	18	16,016	9,333	27,611
6	6	18,627	10,802	32,251
7	4	21,238	12,271	36,891
8	2	23,849	13,740	41,531
9	0	/	/	/
10	0	/	/	/

A cumulative risk table was defined on the basis of MBSS Part A and Part B relative risk. The table reports the cumulative risk of post-operative functional deterioration (upper part – Part A) and long-term unfavorable outcome (lower part – Part B) for each point increase in MBSS Part A and B respectively. Number of patients (N) with the given score, odd ratio and 95% confidence intervals are also reported.
